# Cultivating health: millets' potential in combating non-communicable diseases and future research avenues in India

**DOI:** 10.3389/fnut.2023.1190111

**Published:** 2023-09-21

**Authors:** Sudip Bhattacharya

**Affiliations:** Department of Community and Family Medicine, All India Institute of Medical Sciences, Deoghar, Jharkhand, India

**Keywords:** millet, MILLET MISSION, non-communicable disease (NCD), epidemiologic transition, diabetes, hypertension, prevalence, NCD and risk factors

## NCD burden and the role of nutrition

Chronic illnesses, often referred to as non-communicable diseases (NCDs), present a substantial threat to global public health. According to the World Health Organization (WHO), NCDs are responsible for 74% of all global deaths, with cardiovascular ailments, cancer, respiratory disorders, and diabetes standing as the primary culprits (1). Insufficient dietary habits, encompassing diets high in sugar, salt, saturated and trans fats, and lacking in ample amounts of fruits, vegetables, whole grains, and lean sources of protein, pose a notable risk for NCDs. The World Health Organization recommends a diet abundant in fruits, vegetables, whole grains, nuts, and seeds, while avoiding processed foods, sugar, and excessive amounts of saturated and trans fats (2).

A systematic review and meta-analysis have shown that some dietary patterns, such as the DASH diet (3) and the Mediterranean diet (4) can lessen the likelihood of developing NCDs and benefit the treatment of those who already have them (5). These diets encourage the consumption of minimally processed, unprocessed foods and restrict the consumption of processed and high-fat meals. In addition to food, additional lifestyle factors such as physical activity, avoiding cigarettes and excessive alcohol intake, and stress management can aid in the prevention and management of NCDs (6).

Millet-incorporated foods are those that primarily feature millet as their main ingredient. Millet falls within the category of small-seeded grains, widely consumed across the globe, particularly in Asia and Africa (7). Various types of millets exist, including Sorghum (also recognized as Jowar), pearl millet, Finger millet, Barnyard millet, Proso millet, Foxtail millet, Kodo millet, Little millet, Brown top millet, Fonio, and Tef (7) (Table 1).

**Table 1:** Different types of millet.

**Millet**	**Scientific name**	**Common names**	**Major areas for production**	**Use**
Sorghum	*Sorghum bicolor*	Great millet, jowar, cholam, jola, jonna, durra, Egyptian millet, feterita, Guinea corn, jwari, juwar, milo, shallu, gaoliang, kaoliang, kafir corn, dura, dari, mtama, solam.	USA, Nigeria, Sudan, Mexico, Ethiopia, India, Argentina, China, Niger, Australia	Grown for food grain in Asia and Africa, for fodder in Americas
Pearl millet	*Pennisetum glaucum*	Bajra, cattail, bulrush, candlestick, sanyo, munga, seno	India, Western & Central Africa, Eastern & Southern Africa	Grown for food grain in Asia and Africa, for fodder in Americas
Finger millet	*Eleusine coracana*	Ragi, African, bird's foot, rapoko, Hunsa, wimbi, bulo, telebun, koracan, kurakkan	India, Ethiopia, Nepal, Uganda, Malawi, Burundi, Sri Lanka, Rwanda	Grown for food grain and beer making in Asia and Africa
Foxtail millet	*Setaria italica*	Italian, German, Hungarian, Siberian, kangani, navane, thanahal	China, Myanmar, India, Eastern Europe	Grown for food grain and fodder
Proso millet	*Panicum milliaceum*	Common, hog, broom, samai, Russian, panivarigu, panic, maha meneri	Russia, USA, Ukraine, South Korea, Kazakhstan, France, Poland, Belarus, India, Iran	Grown for food grain and bird seed
Little millet	*Panicum sumatrense*	Blue panic, heen meneri	India	Grown for food grain
Kodo millet	*Paspalum scrobiculatum*	Varagu, bastard, ditch, naraka, water couch, Indian paspalum, creeping paspalum, amu	India	Grown for food grain
Barnyard millet	*Echinochola crus-galli*	Japanese, sanwa, sawan, Korean, kweichou	India, Japan, China, Malaysia	Grown for food grain
Tef	*Eragrostis tef*	Abyssinian lovegrass	Ethiopia, Eritrea, Australia	Grown for food grain, and fodder
Fonio	*Digitaria exilis*	Fundi, hungry rice, acha	West Africa, Sudan, Ethiopia, Nigeria, Niger, Togo, Senagal, Mali	Grown for food grain in Africa

Due to the inherent constituents present in millet seeds such as proteins, peptides, polyphenols, polysaccharides, oil, and isoflavones, millet showcases properties that support well-being. While numerous of these health advantages have been confirmed through studies involving animal models and laboratory tests, the available literature pertaining to favorable effects through human intervention trials remains limited (8).

Millet-based foods are available in numerous forms, including millet porridge, which is considered as a popular breakfast meal in many nations, particularly in Africa and Asia. It can be prepared by heating millet grains in water or milk and adding honey, sugar, or fruit for sweetness. Millet flour can be used to make roti, chapati, and naan, among other forms of flatbreads. They are commonly used in Indian cuisine. Millet can be used as a base for casseroles, or it can be combined with vegetables, beans, or meat to create a nutritious and full meal. Millet can be popped like popcorn and seasoned with salt, butter, or spices to create a nutritious and delicious snack. Much research has explored the health advantages of millet-based foods, and the following are the most significant findings.

Incorporating millet-based foods with a low glycemic index (GI) into diet can be beneficial for managing blood sugar levels (9). A study revealed that a low-GI diet is more effective at reducing glycated hemoglobin and fasting blood glucose in individuals with type 2 diabetes when compared to both a high-GI diet and a control diet (10).

In a separate study, an 8-week regimen of millet-based eating contributed to significant weight loss among overweight and obese participants (9). The inclusion of millet in one's diet holds the potential to lower blood pressure due to its naturally low sodium content; research demonstrated a reduction in blood pressure among hypertensive rats fed millet-based diets (9).

Millet-based diets are rich in antioxidants (8), which offer potential for reducing inflammation and providing protection against chronic ailments such as heart disease and cancer. The spectrum of benefits from millet-derived foods includes their antihypertensive, anti-inflammatory, antimicrobial, hypocholesterolemic, hypoglycemic, and anti-oncogenic properties, coupled with their capacity to positively impact gut health through immunomodulation (9). A systemic review suggests that, millet consumption over a period of 21 days to 4 months, there is a significant reduction in total cholesterol (TC), triacylglycerol, high-density lipoprotein cholesterol (HDL-C), low-density lipoprotein cholesterol (LDL-C), and very-low–density lipoprotein cholesterol (VLDL-C). In four investigations it is also observed that millets ingestion has normalized TC and triacylglycerol levels (<200 and <150 mg/dl, respectively). Millet-based meals also increased HDL-C by 6.0%, lowered blood pressure by 4.0 and 5.0%, and lowered BMI by 7.0% (9). Overall, these studies suggest that integrating millet-based items into the diet has the potential to support in the control of metabolic disease. It is crucial to emphasize, however, that millet should be consumed in moderation as part of a well-balanced diet, and persons with metabolic disorders should consult a healthcare provider before making dietary adjustments.

## Mechanism of action

Multiple mechanisms have demonstrated the efficacy of millet-infused diets in the regulation of metabolic conditions like diabetes, obesity, and hypertension. Some of the avenues through which millet-based foods can contribute to the management of these disorders encompass the following:

One of the pathways involves the low glycemic index of millet, which facilitates a gradual and more consistent increase in blood sugar levels compared to high glycemic index foods like white bread and potatoes. This property can empower individuals with diabetes to better control their blood sugar levels. Additionally, millet possesses a notable fiber content, which aids in slowing down digestion and enhancing the sensation of fullness. This, in turn, can assist in weight management and reduce the risk of obesity (11). Millet is an excellent provider of numerous critical elements, such as magnesium, potassium, and vitamin B6 (9). These nutrients contribute to a reduction in blood pressure and an improvement in insulin sensitivity, which are both essential for overall health. Millet is also abundant in antioxidants, which can reduce inflammation and protect against chronic diseases including heart disease and cancer (8, 9, 11).

Millet is inherently free from gluten, rendering it a healthful choice for individuals afflicted with celiac disease or gluten sensitivity, who need to steer clear of grains containing gluten like wheat, barley, and rye. Incorporating millet-based products into one's dietary regimen can serve as an effective strategy for addressing metabolic disorders (12). However, it's essential to underscore the importance of consuming millet in reasonable amounts and as a component of a well-rounded diet. Prior to making any dietary alterations, individuals with metabolic conditions are advised to seek guidance from their healthcare provider or a qualified dietitian.

## Initiatives of the Indian government toward millet-based diet

According to reports from the Ministry of Agriculture and Farmers' Welfare (7), the millet cultivation area witnessed a significant decline of 60 percent during the 2016–2017 period. This decline is attributed to a combination of factors including shifting dietary preferences, reduced demand, and the redirection of irrigated land toward wheat and rice production. Unfortunately, this situation negatively affected women and children, leading to depletion in their stores of vital nutrients such as vitamin A, protein, iron, and iodine. India's position on the Global Hunger Index (GHI) is ranked at 64 out of 81 countries, revealing a disheartening reality where we stand as the second-worst globally in terms of childhood malnutrition (7).

This predicament is unlikely to improve given that the Public Distribution System (PDS) and the Targeted PDS continue to prioritize the distribution of rice and wheat over millet. Despite its significance for both regional and national food security, millets have not been given a high priority status within Indian agriculture. The importance of millet, as highlighted above, is undeniable. The declaration of 2023 as the “International Year of Millets” by the U.N. General Assembly has drawn greater attention to revitalizing millet consumption. The Indian government has taken various measures to promote millet-based diets and enhance their consumption (Table 1). The primary objectives of the MILLET MISSION encompass the following:

“To generate awareness of the contribution of millet to food security and nutrition.

To inspire stakeholders to improve sustainable production and quality of millets. AND

To focus on enhanced investment in research and development and extension services to achieve the other two aims.”

Listed below are examples of these initiatives: In 2023, the Indian government proclaimed 2023 as the “International Year of Millets” to promote the cultivation and consumption of millets throughout the nation (Table 2) (7). The government has started the MILLET MISSION to encourage millet production and increase millet consumption in the country (7). The mission aims to expand the cultivated land dedicated to millet production, encourage agricultural practices centered around millets, and enhance awareness regarding the nutritional advantages associated with these grains. Additionally, across schools nationwide, the government has introduced millet-based lunches as part of its initiatives. The objective is to offer school children with nutritious and healthful meals while boosting the consumption of millets. The government is actively advocating for millet-based products, which encompass items like cookies, snacks, and ready-to-eat meals. These innovations are being developed to cater to the increasing demand for nutritious and health-conscious dietary choices. The Indian government is further facilitating millet cultivation and processing, along with initiatives for product development and marketing research. Notable programs in this regard include the “Integrated Cereals Development Programmes in Coarse Cereals ICDP-CC based Cropping Systems Areas under Macro Management of Agriculture—MMA,” the “Initiative for Nutritional Security through Intensive Millet Promotion—INSIMP,” which is a part of the “Rashtriya Krishi Vikas Yojana—RKVY,” and the “Rainfed Area Development Programme—RADP,” a component of the “Rashtriya Krishi Vikas Yojana—RKVY” (7). These endeavors reflect the government's commitment to fostering healthful and sustainable food systems across the nation. The underlying aim is to endorse the consumption of millets, which present themselves as nourishing, health-conscious, and ecologically responsible culinary options.

**Table 2:** International and national initiatives for millet.

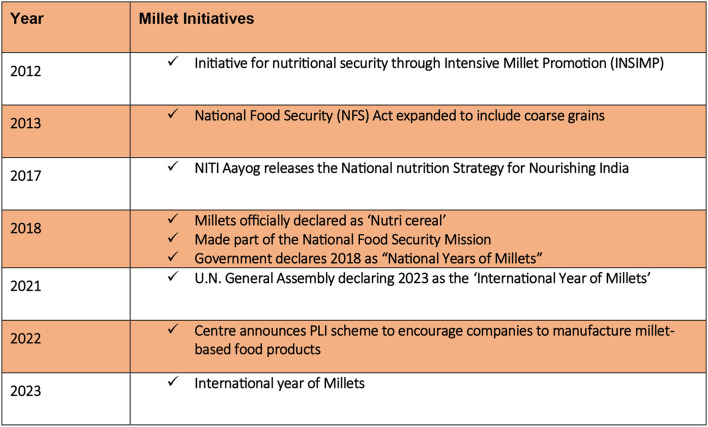

Several other initiatives have also recently been introduced, including the “India's Wealth, Millets for Health” campaign, the “Millet Startup Innovation Challenge,” the “Mighty Millets Quiz,” and a “Logo and Slogan Contest.”

## Future avenues of millet-based food research

In recent times, there has been a surge in the popularity of millet-centric diets, primarily attributed to their promising health benefits and environmentally conscious nature. Delving deeper into this realm through continued research could provide insights into the precise mechanisms driving the health advantages offered by millets, as well as their prospective roles in mitigating and preempting metabolic disorders. These represent promising directions for potential future exploration within the realm of research on millet-based foods.

### Bioavailability

Further inquiry is needed to comprehend the nutritional makeup and effective absorption of elements within millets, encompassing minerals, vitamins, and phytochemicals. Such investigation holds the potential to provide deeper insights into the possible health benefits of millets and their potential utility in addressing deficiencies of essential nutrients.

### Processing and product development

Research efforts can focus on innovating new millet-derived products and refining processing techniques to improve the sensory attributes, shelf life, and nutritional value of these food items.

### Effect on gut microbiota

Research can examine the effect of millet-based diets on the gut microbiota and how this may contribute to their possible health advantages, like curing chronic inflammatory diseases of gut. The efficiency of millet-based diets in controlling, averting, and preventing metabolic illnesses such as diabetes, obesity, and hypertension can be the subject of future research. This can include more translational research from animal to human.

### Sustainability

Research endeavors can delve into assessing the ecological repercussions of millet cultivation and processing, along with investigating the societal implications of endorsing agricultural and food systems centered around millets. Despite the evident support for millets' potential benefits in existing literature, numerous studies exhibit methodological shortcomings, such as reliance on observational designs, limited sample sizes, and challenges in generalizing findings. A notable proportion of these published sources are based on animal studies, which inherently hinder their applicability to broader human contexts. Consequently, an increased focus on human-based interventional research concerning millet-infused foods could significantly contribute to establishing a robust and sustainable food ecosystem.

### Health benefits and metabolic impact

Conducting comprehensive studies to ascertain the specific mechanisms through which millets contribute to managing metabolic disorders such as diabetes, obesity, and hypertension, thereby enhancing their application in preventive and therapeutic interventions.

### Social and economic implications

Exploring the socioeconomic implications of promoting millet-based agricultural and food systems, including their potential to address food security, support local economies, and improve livelihoods.

### Clinical trials on humans

Conducting well-designed intervention studies on human subjects to provide concrete evidence of the health benefits attributed to millet consumption, validating their potential role in enhancing overall well-being.

### Culinary innovation

Experimenting with diverse culinary applications and recipes involving millets to create appealing and versatile food products that cater to modern dietary preferences.

### Consumer acceptance and behavior

Investigating consumer perceptions, preferences, and behavior related to millet consumption to understand the factors influencing their adoption and sustainable integration into diets.

### Public policy and promotion

Evaluating the effectiveness of policy measures, educational campaigns, and initiatives aimed at promoting millet consumption, with an emphasis on driving positive dietary shifts and bolstering food security.

### Long-term health outcomes

Tracking the long-term impact of millet consumption on various health parameters, including chronic disease risk reduction, to establish a comprehensive understanding of its contribution to overall health.

### Global collaboration

Encouraging international collaborations and knowledge-sharing to harness a holistic understanding of millets' potential and facilitate their adoption on a global scale.

Future research endeavors in these areas could significantly contribute to unlocking the full potential of millets as a cornerstone of healthy and sustainable diets.

## Author contributions

The author confirms being the sole contributor of this work and has approved it for publication.
